# P-1283. There’s an uptick!: Increasing Cases of Tick-Borne Diseases Transmitted by *Ixodes scapularis* in New Jersey

**DOI:** 10.1093/ofid/ofae631.1464

**Published:** 2025-01-29

**Authors:** Evelyn Wu, Prince Baffour Tonto, Navaneeth Narayanan, Moulika Baireddy, Mathieu Gerbush, David A Robinson, Thomas Kirn, Ahmed Abdul Azim, John P Mills, Keith S Kaye, Bobby Brooke Herrera

**Affiliations:** Stanford Health Care (current), Rutgers Robert Wood Johnson Medical School (past), Los Altos, California; Rutgers Global Health Institute; Rutgers Robert Wood Johnson Medical School, New Brunswick, New Jersey; Rutgers University Ernest Mario School of Pharmacy & Robert Wood Johnson University Hospital, New Brunswick, NJ; Rutgers, Robert Wood Johnson Medical School, Ashburn, Virginia; Office of the NJ State Climatologist, Rutgers University, New Brunswick, New Jersey; Office of the NJ State Climatologist, Rutgers University, New Brunswick, New Jersey; Rutgers Robert Wood Johnson Medical School, New Brunswick, New Jersey; Rutgers Robert Wood Johnson Medical School, New Brunswick, New Jersey; Rutgers Robert Wood Johnson Medical School, New Brunswick, New Jersey; Rutgers Robert Wood Johnson Medical School, New Brunswick, New Jersey; Rutgers Global Health Institute; Rutgers Robert Wood Johnson Medical School, New Brunswick, New Jersey

## Abstract

**Background:**

Tick-borne disease (TBD) transmission is affected by climate factors, particularly temperature and humidity. Statistical models suggest that Lyme disease cases will increase with the warmer climates and that the season will extend, beginning earlier and ending later. The vector for Lyme disease, *Ixodes scapularis*, also transmits the causative pathogens of babesia and anaplasma. We investigated seasonal trends in diseases transmitted by this vector in NJ over 20 years and their relationship with climate factors.

**Figure 1:**
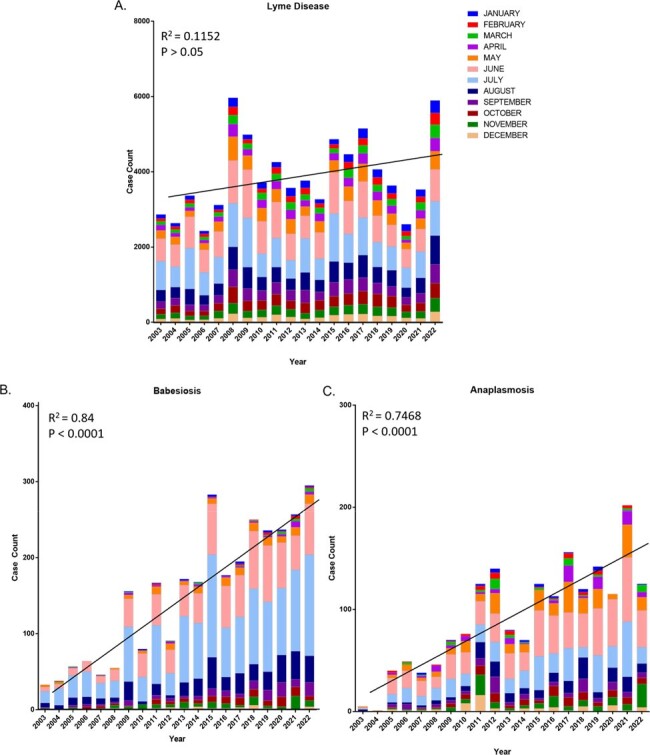
Annual and monthly case counts of (A) Lyme disease, (B) Babesiosis, and (C) Anaplasmosis over the years Annual Lyme Disease cases have not significantly increased. In contrast, annual Babesiosis and Anaplasmosis cases have significantly increased.

**Methods:**

We obtained case counts of Lyme disease, Babesiosis, and Anaplasmosis from 2003-2022 from the NJ Department of Health (N = 83,086) and climate data from the publicly available nClimDiv dataset. We conducted linear regression analysis to assess case count trends over the 20-year period. We performed Poisson regression analysis using case count as the dependent variable, and average temperature and total precipitation as independent variables. For the multivariate Poisson model, we used average temperature and total precipitation as covariates.

**Figure 2:**
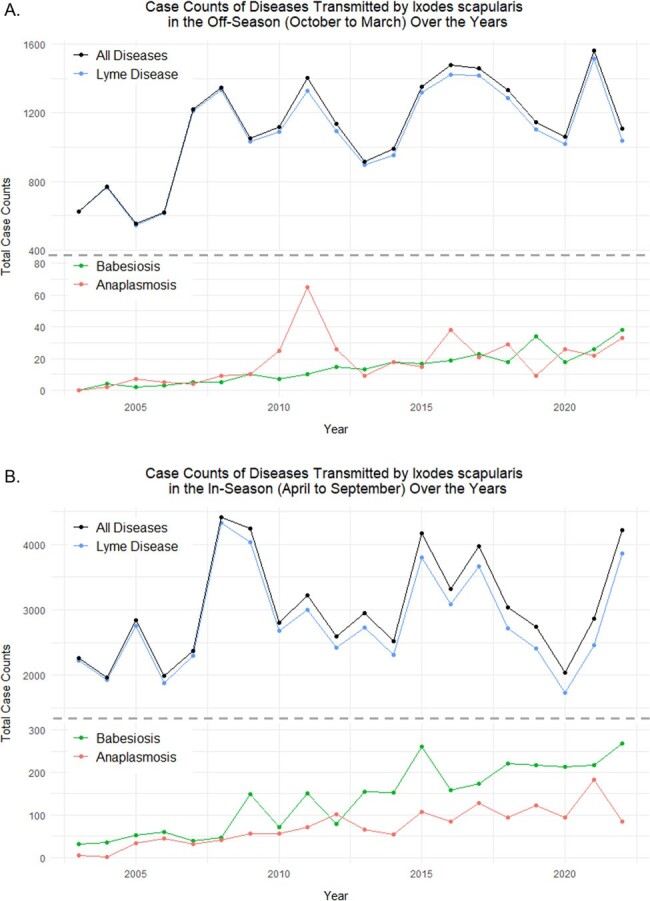
Case counts of Lyme disease, Babesiosis, and Anaplasmosis during (A) Off-Season and (B) In-Season Months over the years (A) Case counts of diseases transmitted by I. scapularis in aggregate and individually have significantly increased in the off-season. (B) Case counts of Lyme disease have not significantly increased in the in-season. In contrast, case counts of both Babesiosis and Anaplasmosis have significantly increased in the in-season.

**Results:**

Lyme disease cases did not significantly increase annually (Fig. 1A). However, seasonal analysis revealed an increase in off-season cases, specifically between December – March (Fig. 2A, p < 0.05). Multivariate Poisson analysis indicated a positive correlation with average temperature only (Table 1).

In contrast, Babesiosis (Fig. 1B) and Anaplasmosis (Fig. 1C) cases significantly increased annually in both the off- and in-season periods (Fig. 2). Babesiosis cases significantly increased in all off-season months (p < 0.01) while Anaplasmosis case counts only significantly increased in October and November (p < 0.01). Multivariate Poisson analysis indicated a positive correlation between average temperature and total precipitation for Babesiosis and Anaplasmosis (Table 1).

**Table 1:**
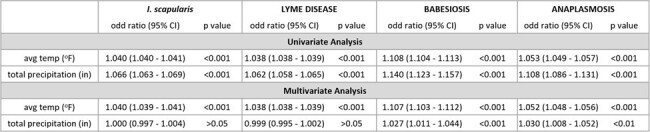
Case count correlation with climate parameters (average temperature and total precipitation) using univariate and multivariate Poisson regression analysis

We performed a Poisson regression analysis using case count as the dependent variable, and average temperature and total precipitation as the independent variables. For the multivariate model, we utilized average temperature and total precipitation as covariates. Total case counts of TBDs transmitted by I. scapularis in aggregate and individually are correlated with average temperature and total precipitation using univariate analysis. In the multivariate analysis, TBDs transmitted by I. scapularis in aggregate and Lyme Disease individually are positively correlated with average temperature while Babesiosis and Anaplasmosis are positively correlated with both climate parameters.

**Conclusion:**

Our findings show that *I. scapularis*-transmitted diseases follow similar trends and are correlated with temperature and precipitation. Lyme disease, Babesiosis, and Anaplasmosis cases are increasing in the off-season, suggesting that the TBD season is extending beyond traditional in-season months and emphasizing the need to maintain vigilance for these diseases during traditionally low-risk off-season months.

**Disclosures:**

**Navaneeth Narayanan, PharmD, MPH, BCIDP**, Astellas: Honoraria|Beckman Coulter: Honoraria|Merck: Grant/Research Support|Shionogi: Grant/Research Support **Thomas Kirn, MD PhD**, Selux Diagnostics: Advisor/Consultant|Selux Diagnostics: Honoraria **Keith S. Kaye, MD, MPH**, Allecra: Advisor/Consultant|CARB-X: Advisor/Consultant|GSK: Advisor/Consultant|Merck: Advisor/Consultant|Shionogi: Advisor/Consultant|Spero: Advisor/Consultant **Bobby Brooke Herrera, PhD**, Mir Biosciences, Inc.: co-founder

